# Ethnozoological and commercial drivers of the pangolin trade in Benin

**DOI:** 10.1186/s13002-021-00446-z

**Published:** 2021-03-23

**Authors:** Stanislas Zanvo, Sylvestre C. A. M. Djagoun, Fortuné A. Azihou, Bruno Djossa, Brice Sinsin, Philippe Gaubert

**Affiliations:** 1 Laboratory of Applied Ecology, Faculty of Agronomic Sciences, University of Abomey-Calavi, 01 BP 526, Cotonou, Benin; 2 Laboratoire Evolution et Diversité Biologique (EDB), CNRS/UPS/IRD, Université Toulouse III Paul Sabatier, Bâtiment 4R1, 118 route de Narbonne, 31062 Toulouse cedex 9, France; 3 Laboratoire de Foresterie et de Conservation des Bioressources (LaFCBio), Ecole de Foresterie Tropicale, Université Nationale d’Agriculture, Kétou, Benin; 4 Centro Interdisciplinar de Investigação Marinha e Ambiental (CIIMAR), Universidade do Porto, Terminal de Cruzeiros do Porto de Leixões, Av. General Norton de Matos, s/n, 4450-208 Porto, Portugal

**Keywords:** Ethnozoological knowledge, Spiritual use, Traditional medicine market, Wildlife trade, Pangolins, Benin, West Africa

## Abstract

**Background:**

Pangolins are trafficked in unsustainable volumes to feed both local and global trade networks for their meat and the medicinal properties of their derivatives, including scales. We focus on a West African country (Benin) to assess the medicinal and spiritual values of pangolins among different ethnic groups and identify the cohort of buyers involved in the pangolin trade and related economic values along the chain, notably from local diasporas.

**Methods:**

We organised 54 focus groups in villages surrounding occurrence habitats of pangolins across Benin and conducted 35 individual interviews with vendors from five major traditional medicine markets (TMMs). Our questionnaire addressed the different uses of pangolins, the commercial value of pangolin items, the categories of clients and the related selling prices.

**Results:**

Pangolin meat was strictly consumed as food. Scales, head, bones, tongue, blood, heart and xiphisternum were the items used by local communities as part of medicinal (65% of the focus groups) and spiritual (37%) practices. Scales were the most frequently used item (use value index = 1.56). A total of 42 medicinal and spiritual uses, covering 15 International Classification of Diseases (ICD) categories, were recorded among ethnic groups. The ICD and spiritual categories-based analyses of similarity showed a partial overlapping of ethnozoological knowledge across Benin, although knowledge was significantly influenced by ethnicity and geographic location. The pricing of pangolins both varied with the category of stakeholders (local communities vs. stakeholders of TMMs) and clients (local and West African clients vs. Chinese community) and the type of items sold. The Chinese community was reported to only buy pangolins alive, and average selling prices were 3–8 times higher than those to West African clients.

**Conclusions:**

Our results confirm that pangolins in Africa are valuable and versatile resources for consumption and medicinal / spiritual practices. The pangolin trade in Benin is based on an endogenous and complex network of actors that now appears influenced by the specific, high-valued demand from the Chinese diaspora. Further investigations are required to assess the growing impact of the Chinese demand on the African wildlife trade.

**Supplementary Information:**

The online version contains supplementary material available at 10.1186/s13002-021-00446-z.

## Introduction

Bushmeat—i.e. the wild game from the tropics—constitutes the main animal protein and income sources for rural people in sub-Saharan Africa [1]. In the Congo Basin, individual consumption amounts several dozens of kg per year (e.g. [2, 3]). Bushmeat often represents the cheapest animal protein alternative for poor rural households [4]. Bushmeat hunting also stands among the prime income-generating activities in rural areas of tropical Africa, where the bushmeat trade can generate more than 500 USD per year for a single household hunter (e.g. [5]).

Bushmeat species also play a vital role in traditional African medicine where animal-derived body parts (items) are used for the treatment of diseases, ailments and spiritual purposes (e.g. [6, 7]). The specific markets, mostly urban, where such items are sold add to the bushmeat selling network already connecting rural to main urban centres [8]. As a consequence, bushmeat consumption and use, which occur at unsustainable rates in Africa [9], have so far remained an intractable issue, contributing at the same time to household wealthiness and biodiversity extinction [10, 11].

Pangolins (Pholidota, Mammalia), or scaly anteaters, have recently emerged as the flagship taxon of the bushmeat crisis. They are trafficked in unsustainable volumes to feed the local and international—mostly driven by the traditional Chinese medicine (TCM)—demands for both their meat and the medicinal properties of their scales and other items [12–14]. Pangolins have also been suggested as the intermediate host responsible for the COVID-19 pandemic [15], despite the lack of concrete evidence for this claim. In Africa, pangolins have recently seen their trafficking volumes and market prices increase, in line with the trends observed for the Asian species [16]. Effective trading networks are now connecting Africa and Asia to feed the TCM demand for pangolin scales [14].

Among the four species of African pangolins, the white-bellied pangolin (*Phataginus tricuspis*) is the most frequently found on the bushmeat and traditional medicine markets (e.g. [17, 18]), notably in West Africa [6, 7, 16, 19]. Ethnozoological knowledge on the species shows a diversity of uses by local communities involved in medicinal and spiritual practices, to treat convulsion, rheumatism, hiccups, healing wounds, woman unfaithfulness and impotence [18, 20]. Scales are the most commonly used, although various items such as tongue, bones and head are also regularly found on the traditional medicine market (TMM [6, 7];).

Benin, situated in the Dahomey Gap (West Africa) and often designated as the cradle of the ‘Vodoun culture’, harbours a vibrant market network for animal-based medicinal and spiritual practices, likely to have deleterious impacts on biodiversity conservation in the whole sub-region [6, 7]. Although the ethnozoology of pangolins has received much attention in neighbouring countries [6, 17, 20–22], the situation in Benin, where the white-bellied pangolin’s range has been contracted by 1/3 over the last two decades [23], remains understudied. Akpona et al. [24] found seven different items of pangolins used by southern communities for 13 medicinal and spiritual purposes, with scales as the most frequently cited. However, this study was restricted to southern Benin and included only two ethnic groups (*Hôli* and *Fon*), despite the larger extent of pangolin’s distribution in Benin [23].

Investigating on the ethnozoology of pangolins should help understand the causes and extent of the species decline as related to the medicinal and spiritual practices that prevail in Benin. In this study, we propose a country-scale survey of the main ethnozoological drivers of the pangolin trade encompassing four major Beninese ethnic groups and incorporating the recently raised issue of the international demand from local diasporas [14]. Our main objectives are to (i) assess the different uses of pangolin items among ethnic groups in Benin and (ii) identify the cohort of buyers involved in the pangolin trade and related economic values along the chain, including the local demand from the Chinese community.

## Methods

### Study area

**Fig. 1:**
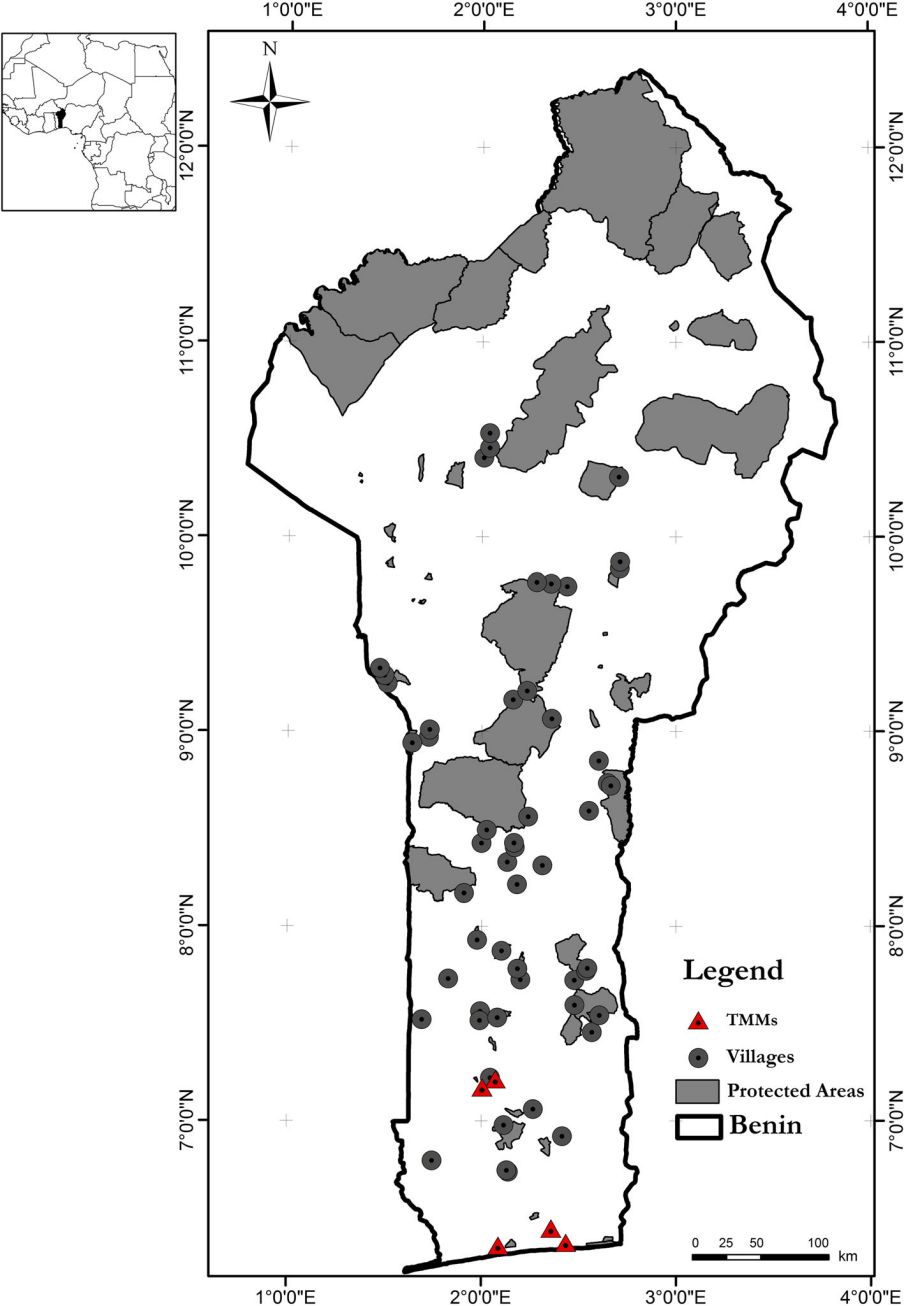
Villages and traditional medicine markets (TMMs) in Benin surveyed as part of this study

The study took place in Benin from April 2018 to April 2019. Benin is a West African country located between latitudes 6° 10′–12° 25′ N and longitudes 0° 45′– 3° 55′ E (Fig. 1). Its vegetation is marked by a severely fragmented forest cover due to both drier climatic conditions during the Holocene [25] and ever-growing human (mostly agricultural) activities [26, 27]. Benin is widely known for its Vodoun culture, which is strongly linked to a diversity of traditional practices (medicinal and spiritual) using various animal derivatives [6, 7]. Vodoun practices have driven the development of a dense TMM (also called ‘fetish markets’) network in the country, with at least 42 markets identified in southern and central Benin (SZ, unpubl. data). Benin counts around 60 ethnic groups for an estimated population of c. 10 M inhabitants [28]. The ethnic group ‘*Fon*’ is dominant in the South whereas ‘*Nagot*’ and ‘*Bariba*’ are the most represented ethnic groups in central and northern Benin, respectively [29].

### Data collection

We targeted (i) 54 villages neighbouring all the potential areas of occurrence of pangolins in Benin and (ii) the five major TMMs in southern and central Benin (*Avogbannan*, *Calavi*, *Dantokpa*, *Gbèdagba* and *Zobè*) (Fig. 1). The target local communities were identified after Zanvo et al. [23] from occurrence areas providing pangolin scales. Focus group participants and vendors were adults at least 18 years old. Focus group participants were volunteers, identified through informal interviews with villagers whereas in the TMMs, availability and trust towards the interviewer were the criteria of participation. In each village, we organized a single focus group with 7–11 local people using a pre-established questionnaire and a poster figuring pangolins. The questionnaire was conducted as a semi-structured interview addressing the names used for pangolins in local languages, the different uses of pangolins and which items were involved, the selling prices of pangolins, the categories of clients and the time period at which the trade with each category of clients started (Additional file 1). For each question, all the answers were recorded without discrimination among participants of the focus group. In the TMMs, we generally had to use a modified approach focusing on the questions related to the commercial value of pangolins. Such a strategy was necessary because we were not able to gather simultaneously several vendors and the latter were not available for long-time interviews, due to their activity. However, six interviewees out of 35 were able to fully answer the questionnaire. Their responses on the ethnozoology of pangolins were treated as a single focus group, as they all belonged to the same *Fon* ethnic group.

### Data analysis

We used the International Classification of Diseases (ICD-11; version 09/2020) to group the recorded medicinal uses into ICD categories for each item of pangolins. Given the important number of spiritual uses recorded, we created an additional, dedicated category. Each focus group was considered as a single observation for the analysis. Descriptive statistics were used to assess the frequency of citation of the items used, ICD categories mentioned by focus group participants and type of clients buying pangolin items. We used the use value index (UV; adapted from [30]) to assess the spectrum of use of each pangolin item for medicinal and spiritual purposes, as follows:

$$ UV=\frac{\sum {U}_P}{n} $$

where *Up* represents the number of uses mentioned by focus groups for each pangolin part and *n* the total number of focus groups.

One-way analysis of similarities (ANOSIM test) based on 9999 permutations was carried out using the *vegan* package in *R* version 4.0.0 [31] on a matrix of ICD and spiritual categories including the ethnic groups partitioned into geographic regions (South, Centre and North Benin). The *R* statistics was interpreted according to Clarke [32]: (i) *R* < 0.25 means no separation between groups, (ii) 0.25 < *R* < 0.5 some level of separation between groups despite a degree of overlap, *R* > 0.75 well separated groups, and *R* = 1 total separation between groups. The ANOSIM test was used to assess variation of knowledge among ethnic groups (recorded at least four times: *Bariba, Fon, Mahi* and *Nagot*) and geographic regions using medicinal and spiritual use patterns. We used non-metric multidimensional scaling (NMDS) analysis to visualize in a Cartesian space the dissimilarity between ethnic groups and geographic regions. We removed meat from ANOSIM and NMDS analyses due to the absence of medicinal and spiritual uses for this specific pangolin item. We calculated the average selling prices among local communities and in the TMMs for the different categories of clients. We used the *t* test to compare average selling prices according to the categories of clients in rural areas (local communities) and in TMMs and between rural areas and TMMs.

## Results

**Fig. 2:**
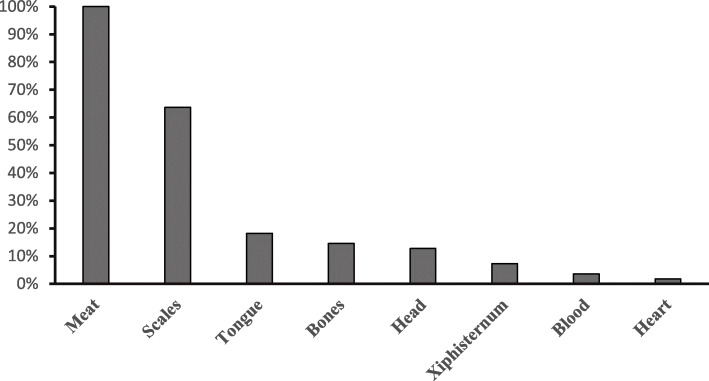
Frequency of citations of pangolin items used by local communities

**Fig. 3:**
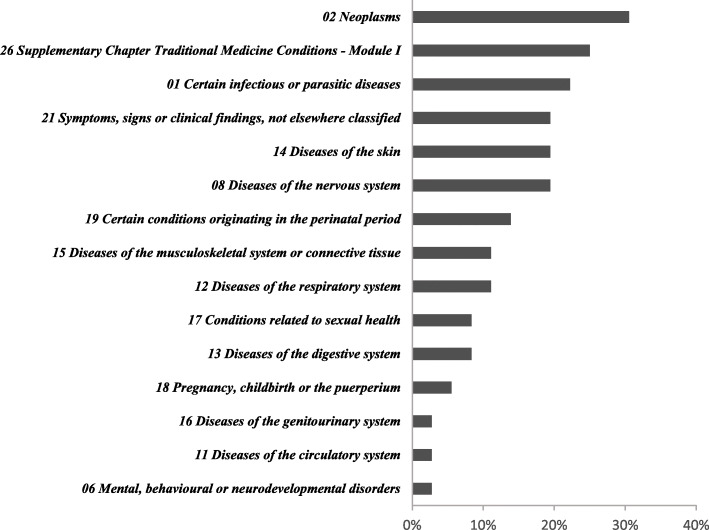
Frequency of citations of the conditions treated with pangolin items according to ICD categories

**Fig. 4:**
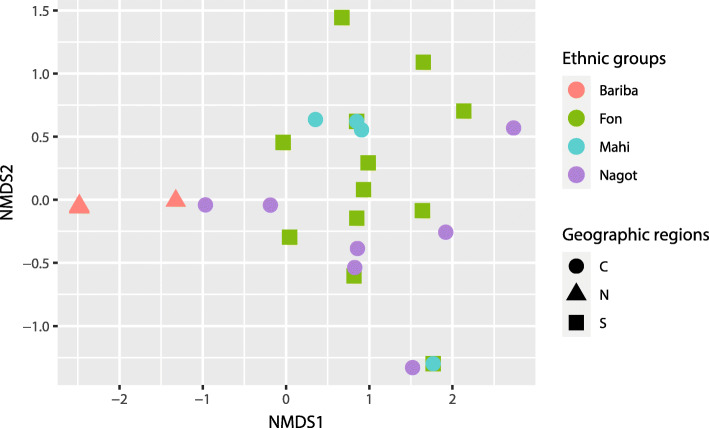
Spatial distribution of ethnic groups according to their ICD and spiritual categories-based knowledge. Euclidean distances between ethnic groups and geographic regions (C—centre; N—north; S—south) reflect the degree of similarity of knowledge. Overlapping implies the sharing of the same knowledge

Fifty-four focus groups were carried out across the occurrence zone of pangolins in Benin with 18, 22 and 16 focus groups in southern, central and northern regions respectively. Thirty-five individual interviews were performed in the TMMs. Focus group participants were mostly men (84%), between 22 and 76 years (mean = 36 years), and included farmers (36%), farmer-hunters (54%) and housewives (8%), whereas all the interviewees from the TMMs were men, aged 26 to 54 (mean = 37). *Lihoui* was the common name almost unanimously used for pangolins among ethnic groups in southern Benin whereas in central Benin, *Nagot, Ifè* and *Idatcha* named pangolins either *Aïka* or *Akikan*. In northern Benin, the common name of pangolins changed from an ethnic group to another (Table 1). Pangolins were unanimously cited by all the focus group participants for their use as an animal protein source. Medicinal and spiritual uses were cited by 65% and 37% of the focus groups, respectively. Scales, tongue, head, bones, xiphisternum (a specific prolongation of the sternum to attach the tongue muscles), blood and heart were the eight pangolin items cited by focus group participants (Fig. 2). Scales (64%), tongue (18%), bones (15%) and head (13%) were the items most cited by the focus groups for medicinal and spiritual uses. A total of 42 medicinal (*n* = 31) and spiritual (*n* = 11) uses were recorded. The medicinal uses fell into 15 out of 26 ICD categories. The scales were the item of pangolins that had the highest use reported (56), number of ICD categories (14) and use value (UV = 1.56), followed by tongue, bones, head, xiphisternum, blood and heart (Table 2). The spiritual use category was the most mentioned by focus group participants (69%), superior to any ICD categories (Fig. 3). Sexual bewitchment was the spiritual use most frequently reported by focus groups, followed by the control of women’s infidelity. Among the 15 recorded ICD categories, neoplasms (ICD-2; 31%), traditional medicine conditions (ICD-26; 25%), and certain infectious or parasitic diseases (ICD-1; 22%) were the most cited ICD categories by focus group participants (Fig. 3). The ANOSIM multivariate analyses showed slight differences of ethnozoological knowledge among the four major ethnic groups (*R* = 0.55; *p <*0.001) and geographic regions (*R* = 0.48; *p <*0.001). All the *R* statistic values ranged between 0.25 and 0.5, indicating that the ethnic groups shared some level of ethnozoological knowledge. The NMDS plot showed some variation of the distance within and between *Fon*, *Nagot* and *Mahi* ethnic groups located in southern and central (Fig. 4). Conversely, the *Bariba* were close to each other, and generally remote from and opposite to all the *Fon*, *Mahi* and almost all the *Nagot*. We observed some overlapping between focus groups among *Bariba*, *Mahi* and *Nagot* ethnic groups. Moreover, some focus groups among *Mahi* and *Nagot* ethnic groups overlapped with *Fon* whereas others were remotetly distributed in space.

**Table 1:** Common names used for the white-bellied pangolin among ethnic groups and geographic regions in Benin

Region	Ethnic groups	Local names
South	*Adja*, *Agouna*, *Aïzo*, *Gun*	*Lihoui*
	*Fon*	*Lihoui*, *Houékin*
	*Yoruba*, *Hôli*	*Iwô*
Centre	*Mahi*	*Kosso Lihoui*
	*Nagot*, *Ifè*, *Idatcha*	*Aïka*, *Akikan*
North	*Anii*	*Sassabokourou*
	*Bariba*	*Kokowaka*
	*Nagot*	*loufiloufi*
	*Lokpa*	*Libilibi*
	*Kotokoli*	*Kaminakpara*
	*Ditamaré*	*Tempétakpara*
	*N'tcha*	*Akonkon*
	*Yom*	*Narm*

**Table 2:** Pangolin’s items used in traditional medecine and spiritual practices in Benin. Spiritual purposes are in bold. Use report (UR), International Classification of Diseases (ICD) and Use Value (UV)

Items	Conditions treated	UR	ICD	UV
**Scales**	**Physical strength**, oedema, cough, hiccups, rheumatism, convulsion of new born, pharyngitis, healing wound, snake bite, measles, **man dominance in household**, healing fire burns, **defeating the opponent in case of litigation**, facilitate delivery, infertility, scabies, **sexual bewitchment**, ringworm, joint pain, stomach aches, vigour of new born, breast cancer, dizziness, heart palpitations, late infantile spinal muscular atrophy, osteoarthritis and asthma	56	14	1.56
**Tongue**	Stomach aches, pneumonia, hip pain, developmental language disorders, **protection against sexual bewitchment**, Epilepsy, **incantation**, **defeating the opponent in case of litigation**, sterility, **thief sickness and stop women’s infidelity**	12	7	0.33
**Bones**	Hiccups, cough, osteoarthritis, healing wound, ataxia, asthma, coxarthrosis, developmental language disorders and late infantile spinal muscular atrophy.	11	6	0.31
**Head**	Oedema, pharyngitis, epilepsy, **defeating the opponent in case of litigation**, vigour of new born, **unnatural power and luck**	9	5	0.25
**Xiphisternum**	**Impotence**, **sexual bewitchment**	4	2	0.11
**Blood**	Asthma and convulsion of new born	3	2	0.08
**Heart**	**Man dominance in household**	1	1	0.03

**Fig. 5:**
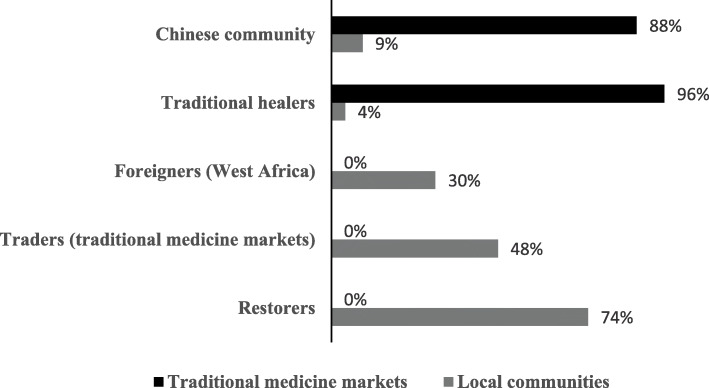
Citation frequencies of client categories within traditional medicine markets (black bars) and local communities (grey bars) in Benin. West African foreigners originate from Ghana and Togo

Local communities cited five client types involved in the trade of pangolins in Benin (restorers [i.e. managers of restaurants], traditional healers, traders of traditional medicine markets, foreigners from West African countries and Chinese community), while only two client types (traditional healers and Chinese community) were reported by TMM stakeholders (Fig. 5). Restorers, traders from TMMs and foreigners from West Africa (Togo and Ghana, according to focus group participants) were cited by 74, 48 and 30% of the focus groups, respectively, as clients buying pangolins to local communities. Traditional healers (4%) and Chinese community (9%) were the client categories less cited by local communities. Within the TMM stakeholders, traditional healers (96%) and Chinese community (88%) were the only clients cited as buying pangolins. Local communities and the TMM stakeholders highlighted that the trade of pangolins with local people exists since the development of wildlife markets whereas the trade with West African and Chinese communities started approximately one decade ago. Pangolin pricing changed according to the category of stakeholders (local community *vs*. trader in TMMs) and clients (local and West African clients *vs*. Chinese community) (Table 3). The average selling prices were higher in the TMMs than in local communities. The Chinese community bought only pangolins alive, while local and West African customers both bought alive and dead pangolins or pangolin items. Head and scales were only sold in the TMMs to local and West African clients. The highest selling price (73.38 USD) for a pangolin (alive) was recorded in a TMM for a client from China. The average selling price of pangolins sold on the TMMs to the Chinese community was significantly higher (*t* = -34.089; *p* < 0.001) than the selling prices to local and West African clients. The same trend (*t* = − 16.238; *p* < 0.001) was recorded at the local community level with the Chinese community buying pangolins alive at higher prices than local and West African clients. There was no significant difference between the average selling prices to Chinese community in local communities and TMMs.

**Table 3:** Variation in pangolin pricing between local communities and traditional medicine markets in Benin, after client categories. African clients other than local originate from Togo and Ghana. Prices are given in USD (conversion: 30 July 2020). TMMs refers to traditional medicine markets

		Pangolin pricing (USD)			
		Local communities		TMMs	
Type of item	Average	Local and West African clients	Chinese clients	Local and West African clients	Chinese clients
**Alive**	Min	5.01	42.9	18.96	55.35
	Max	7.84	60.05	24.83	73.38
**Dead carcass**	Min	4.52	–	18.96	
	Max	6.68	–	24.83	
**Head**	Min	–	–	3.88	
	Max	–	–	4.73	
**Scales (whole body)**	Min	–	–	8.62	
	Max	–	–	12.06	

## Discussion

Relative to the sole study that had been conducted on the ethnozoology of pangolins in southern Benin [24], our investigations provide a deeper understanding of ethnozoological values across a diversity of ethnic groups in combination with the economic incentives possibly motivating the overexploitation of pangolins in Benin [23].

*Lihoui* was the common name for pangolins among most of the ethnic groups in southern Benin, whereas *Aîka*/*Akika* was used in central Benin. This uniformity of common names among ethnic groups located within the same geographic region (*Fon*, *Aizo*, *Adja* and *Gun* in South, and *Nago*, *Ifè* and *Idatcha* in Centre) could be due to the genealogical relationships between the sampled ethnic groups, which originate from the same ancestors and share similar languages [29]. The *Fon* ethnic group is believed to be the initiator of the trade in animal derivatives since the Dahomey Kingdom (from c. 1600 to 1904 AD) and represents the vast majority of the actors currently operating in this sector in Benin (SZ, unpubl. data). The common name *Lihoui* could have originated from this dominant ethnic group and through the colonization of other ethnic groups have diffused since centuries in all the TMMs of southern Benin (see [33]). Conversely, northern Benin showed eight different names used for pangolins. This is likely because (i) this region has been colonized by ethnic groups from different origins with socio-linguistic divergences [34, 35] and (ii) the TMM network is almost inexistent, thus preventing from any diffusion of a dominant pangolin name.

Pangolins are used for food, medicinal and spiritual purposes in Benin, in line with the literature record for tropical Africa [21, 22, 36–41]. More specifically, pangolins in Benin constitute at the same time a bushmeat resource and an important input to traditional medicine and cultural practices [24]. Pangolin meat is unanimously consumed as food in Benin, whereas no medicinal and spiritual use is recorded, in line with Akpona et al. [24] but contrary to Boakye et al. [21, 37] and Baiyewu et al. [36] who found that meat was used in traditional medicine in several countries of western and southern Africa.

Among the eight recorded pangolin items, scales were by far the most commonly used by local communities for medicinal and spiritual purposes. Scales had the highest use value (UV = 1.56) and were mentioned in 14 out of the 15 ICD categories recorded for 56 different use reports. These results show that the scales possess a great traditional value for local people in Benin, probably justifying the storage of old scale samples in rural households [23] and the great number of scales present in the TMM stalls (SZ, CD, PG; unpubl. data). Our results corroborate those of previous studies in tropical Africa pointing out the high use value and versatility of use of pangolin scales in comparison with other items [21, 37, 42]. Although the diversity of items for ethnozoological use was generally lower in Benin than in other African countries ([6, 21, 37]; but higher than in [24, 43]), we observed the use of a so far unreported pangolin item, the xiphisternum. The latter is involved in impotence and sexual bewitching treatments and could be a particular knowledge of *Fon* and *Mahi* ethnic groups.

A large proportion of the disease and ailment treatments where pangolin items are involved had never been described for Benin [24], although almost all of them had already been recorded in West Africa [21, 37]. The ratio between the diversity of medicinal/spiritual uses and items was higher in Benin (42 medicinal and spiritual purposes for eight items) than what was recorded from traditional healers and fetish markets in other West African countries [6, 21, 37]. In Benin, spiritual uses (69%) and the ICD categories neoplasms (31%) and traditional medicine conditions (25%) were the most cited by local communities. Overall, this indicates that pangolin items are used against many diseases and ailments generally uncovered by conventional medicine, revealing the high endogenous value of pangolins in a country where Vodoun practices are thriving [44].

Our results suggested slight differences of ethnozoological knowledge within and between ethnic groups and geographic regions, and at the same time indicated partial overlapping of ethnozoological knowledge among the major ethnic groups (*Fon*, *Nagots*, *Bariba* and *Mahi*) in Benin. Socio-cultural distances between ethnic groups in Benin (see [34, 35]) could be a factor explaining the divergences of knowledge on medicinal and spiritual uses of pangolins. For instance, the use of scales to treat breast cancer was only cited by the *Bariba* ethnic group whereas pneumonia, epilepsy, rheumatism and measles were only recorded among *Fon*. The *Nagot* were the only ethnic group that mentioned the use of pangolin items for the treatment of ringworm, scabies and heart palpitations. Thus, our results show a partial influence of ethnicity on ethnozoological knowledge of pangolins, in line with what was observed in Ghana [20]. However, *Fon*, *Mahi* and *Nagot* ethnic groups also share ethnozoological knowledge on pangolins such as healing wound, treatment of sterility, hiccups, easy delivery, snakebite, pharyngitis, sexual bewitchment, defeating the opponent in case of litigation, and sterility. South and central Benin count 42 TMMs (SZ, unpubl. data) mainly dominated by the *Fon* ethnic group, whose beliefs are strongly linked to Vodoun practices. Through this market network, the *Fon* ethnic group has probably diffused or gained medicinal and spiritual knowledge of pangolins in the central region of Benin.

As part of the bushmeat species spectrum sold in Central and West Africa, pangolins constitute a traditional source of income for rural communities, restorers and TMM vendors [6, 16, 19]. In Benin, we showed that the pricing of pangolins both varied with the category of stakeholders (local communities *vs*. stakeholders of TMMs) and clients (local and West African clients *vs*. Chinese community) and the type of items sold (from alive to scales). Average selling prices for live and dead pangolins were higher in TMMs (18.96–73.38 USD), probably to write off the costs of being at the tip of the supply chain and to benefit from the higher wealthiness of urban households [4]. However, excluding buyers from the Chinese community, average selling prices of pangolins in rural areas (4.52–7.84 USD) and in TMMs (18.96–24.83 USD) were lower than those recorded in the bushmeat markets from central Africa one decade ago (mean = 34.27–35.66 USD [14]). The relatively cheap cost of pangolins on the Beninese markets means that the species is economically reachable by West African consumers, which may not facilitate the mitigation of the volumes of pangolins extracted each year in Benin as part of the wildlife trade.

Our study highlighted the international component of the pangolin trade in Benin through the diversity of foreign clients, involving West African and Chinese nationals since c. one decade (probably also involving Vietnamese nationals, after a case of recent seizure at Cotonou airport [14]). Whether pangolins are consumed locally or brought back to the clients’ home countries is unknown and would deserve further investigations. We revealed different practices according to the type of clients, the Chinese community only buying pangolins alive whereas local and West African clients would also buy dead pangolins and various items (head, scales). The average selling prices of pangolins to the Chinese community was 3–8 times higher than the selling prices to local and West African clients, confirming that Chinese diasporas in Africa has brought further economic incentives to the endemic pangolin trade [16, 19]. Further investigations are required to assess whether the Chinese diaspora demand has modified the practices of wildlife traders and is constitutive of an ‘overexploitation vortex’ of pangolins (see [45]) in Benin.

Assuming a mean body weight of 2.48 kg for the white-bellied pangolin (2.36 ♂–2.6 ♀ kg; in [46]) and a 1/4–1/3 contribution of the scales to the total weight of the species [47], scales bought by local and West African clients would cost 10.39–19.45 USD per kilogramme. Because there is a possibility that the scales of pangolins bought by the Chinese community in Benin end up feeding the global pangolin trade (e.g. 513 kg of scales were seized in Cotonou in 2018 [14]), we also estimated the average price of pangolin scales per kilogramme if scales were extracted from live pangolins (17.30–29.59 USD per kg). Such prices confirm that pangolin scales constitute a valuable source of income on West African markets [21]. Compared to the prices of pangolin scales sold in China and Vietnam (485–759 USD per kg) [12], both paths of acquiring scales in Benin (either directly or from a live animal) would remain highly profitable to traffickers that feed the illegal pangolin trade to—mostly—China [48].

Studying client practices allowed us to delineate a complex network for the pangolin trade in Benin, including (i) the TMM that mostly supplies traditional healers and the Chinese community and (ii) a less urbanized and more diffused market network where local and West African clients, together with restorers and TMM stakeholders, would be the main buyers. The low contribution of the Chinese community as mentioned by local communities (9%; only from the Lama forest reserve, the main forest island from southern Benin) may imply that intermediates collect pangolins from more proximal sources than the TMMs, but this will require further investigations.

## Conclusions

Our study addressed the ethnozoology of pangolins across Benin and its major ethnic groups and revealed the importance of the traditional (medicinal and spiritual) and economic values of pangolins for local communities and TMM vendors. Our results suggest that the pangolin trade in Benin is based on endogenous practices now influenced by economic drivers (higher prices and change of selling practices) from the local Chinese demand and that a number of actors are involved in an intricate, multi-scale network. Conserving pangolins in Benin will require considering the multiple, cultural and economic drivers of the market and engaging synergic efforts against both endemic and international trafficking. Long-term monitoring of offtake and trafficking network in Beninese markets and targeted habitats, together with higher law enforcement, will be essential to reverse the decline of the white-bellied pangolin as witnessed by Beninese rural communities [23].

## Supplementary Information

**Additional file 1.** Questionnaire sur l’ethnozoologie et le commerce des pangolins.

## Data Availability

All the data generated and analysed during the current study are included in this manuscript.

## Abbreviations

ANOSIM: Analysis of similarity
ICD: International Classification of Diseases
NMDS: Non-metric multidimensional scaling
TMM: Traditional medicine market
TCM: Traditional Chinese medicine
UR: Use report
USD: United State dollar
UV: Use value
