# Supplementary material for: Ethnozoological and commercial drivers of the pangolin trade in Benin
Source: J Ethnobiol Ethnomed. 2021 Mar 23;17:18. doi: 10.1186/s13002-021-00446-z (PMC7985750; doi:10.1186/s13002-021-00446-z)
Supplement: Supplementary file 1 — Additional file 1. Questionnaire sur l’ethnozoologie et le commerce des pangolins. [file 13002_2021_446_MOESM1_ESM.docx]

**Additional file 1**

**Questionnaire sur l’ethnozoologie et le commerce des pangolins**

Je m’appelle Stanislas Zanvo, Doctorant au Laboratoire d’Ecologie Appliquée, Université d’Abomey-Calavi. Je travaille sur l’espèce que vous voyez sur ce poster. Une partie de mes travaux consiste à investiguer les valeurs ethnozoologique et commerciale du pangolin à travers son aire de répartition au Bénin. Ce travail est purement scientifique, et la participation aux discussions est volontaire et facultative. Cependant, nous garantissons à tous les participants l’anonymat et leur donnons toute notre assurance. Les informations que vous nous fournirez ne seront pas utilisées pour causer des dommages à un participant, ni votre communauté.

**I. Identité des participants**

1. Nombre: Hommes…………… Femmes………..

2. Groupe ethnique: ……………………………………………………………………………..

3. Profession et âge des participants:

| **Profession** | **Nombre de participants** | **Age** | **Niveau d’éducation** |
| --- | --- | --- | --- |
|  |  |  |  |
|  |  |  |  |
|  |  |  |  |
|  |  |  |  |
|  |  |  |  |
|  |  |  |  |
|  |  |  |  |
|  |  |  |  |
|  |  |  |  |

1. **Connaissance sur les pangolins**

4. Connaissez-vous les espèces animales sur le poster (montrer)? Oui Non

5. Confirmez-vous l’existence d’au moins une de ces espèces dans votre milieu? Oui Non

6. Citer les habitats de ces espèces (˂ dans un rayon de 3 km) dans votre milieu. ……………………………………………………………………………………………………………………………………………………………………………………………………………………………………………………………………………………………………....

7. Quels sont les noms locaux de ces espèces animales (dans votre groupe ethnique uniquement)? ................................................................................................................................

.......................................................................................................................................................

8. Existe-t-il des interdits liés à ces espèces dans votre communauté (groupe ethnique)?

Oui Non

Si oui, citez-les ……………………………………………………………………………………………..

……………………………………………………………………………………………………………………………………………………………………………………………………………………………………………………………………………………………………………………………………………………………………………………………………………………………………………………………………………………………………………

1. **Ethnozoologie des pangolins**

9. Les pangolins sont-ils des animaux utiles pour vous? Oui Non

10. Si oui, citez toutes les formes d’utilisation de ces animaux dans votre communauté.

| **Partie de l’animal** | **Usage** |
| --- | --- |
|  |  |
|  |  |
|  |  |
|  |  |
|  |  |
|  |  |
|  |  |
|  |  |
|  |  |
|  |  |
|  |  |
|  |  |
|  |  |
|  |  |
|  |  |
|  |  |
|  |  |
|  |  |
|  |  |
|  |  |
|  |  |
|  |  |
|  |  |
|  |  |
|  |  |
|  |  |

11. Sinon, quelles sont les raisons? .....................................................................................................................................................

………………………………………………………………………………………………………………………………………………………………………………………………………………………………………………………………………………………………………………………………………………………………………………………………………………………………………………………………………………………………………………………………………………………………………...........................................................

1. **Valeur commerciale et réseau de commerce des pangolins**

12. Les pangolins constituent-ils une source de revenus pour vous dans votre village (les chasseurs par exemple)? Oui Non

14. Si oui, citer les catégories d’acheteurs de pangolin dans votre village

| **Categories d’acheteurs** | **Nationalité** | **Depuis quand sont- ils devenus vos clients?** |
| --- | --- | --- |
|  |  |  |
|  |  |  |
|  |  |  |
|  |  |  |
|  |  |  |
|  |  |  |
|  |  |  |

15. Quelles sont les formes de vente de pangolins dans votre village?

Vivants morts parties du corps

16. Donner les prix de vente d’un pangolin (vivant ou mort), et des parties de l’animal.

| **Items** | **Prix de vente** | | **Types de clients** |
| --- | --- | --- | --- |
|  | min | max |  |
|  |  |  |  |
|  |  |  |  |
|  |  |  |  |
|  |  |  |  |
|  |  |  |  |
|  |  |  |  |
|  |  |  |  |
|  |  |  |  |
|  |  |  |  |
|  |  |  |  |
|  |  |  |  |
|  |  |  |  |
|  |  |  |  |
